# Tadalafil monotherapy in management of chronic prostatitis/chronic pelvic pain syndrome: a randomized double-blind placebo controlled clinical trial

**DOI:** 10.1007/s00345-022-04074-4

**Published:** 2022-07-08

**Authors:** Ahmed M. Tawfik, Mohammed H. Radwan, Mohammed Abdulmonem, Mohammed Abo-Elenen, Samir A. Elgamal, Mohammed O. Aboufarha

**Affiliations:** 1 Urology Department, Faculty of Medicine, Tanta University, Tanta, Egypt; 2 Urology Department, Faculty of Medicine, Zagazig University, Zagazig, Egypt

**Keywords:** Tadalafil, PDE5-inhibitors, Prostatitis, Pelvic pain, CP/CPPS, Prostatic pain, Chronic prostatitis chronic pelvic pain

## Abstract

**Purpose:**

In this placebo-controlled trial, we aimed to evaluate the clinical results of using PDE-5 inhibitor, tadalafil 5 mg OD, for management of CP/CPPS.

**Patients and methods:**

140 patients ≤ 45 years old with moderate/severe CP/CPPS associated with ED (IIEF-5 < 22) were randomly divided and received either tadalafil 5 mg OD (tadalafil-group) or placebo (control-group) for 6 weeks. Post-treatment CPSI scores were compared to baseline and to placebo. Clinically significant responders (≥ 25% reduction from baseline score) were calculated. Tadalafil-induced changes in IIE-5 were evaluated in correlation to that of CPSI scores.

**Results:**

By the 6th week, 59 and 56 patients were available in both groups respectively. Compared to baseline, tadalafil-group patients showed significant improvement in total, pain, urinary and Qol domains of CPSI (19.1 ± 5.26, 10.42 ± 3.55, 4.2 ± 1.72 and 4.47 ± 1.64 vs. 24.21 ± 5.05, 12.14 ± 3.57, 6.08 ± 1.53 and 6.22 ± 1.76), *p* < *0.5*. When compared to placebo, all 6th week CPSI domains scores, except for pain, were significantly better in tadalafil-group (*p* < *0.05*). Post-treatment pain score didn't significantly differ between both groups (10.42 ± 3.55, vs. 11.71 ± 3.9, *p* > 0.05). Clinically significant responders were 30 patients (50.8%) in tadalafil-group vs. 3 patients (5.4%) in control. Tadalafil-induced changes in IIEF-5 score had weak but significant correlation to Qol domain (*r* = − 0.28, *p* < 0.05).

**Conclusion:**

Tadalafil 5 mg OD can significantly improve all CPSI domains as compared to baseline. Post-treatment CPSI scores, except for pain, were better than placebo. About 50.8% of patients can develop ≥ 25% reduction in their total CPSI scores after treatment. Apart from Qol domain, these changes are not significantly correlated to tadalafil-induced IIEF-5 scores changes.

## Introduction

Chronic prostatitis associated chronic pelvic pain syndrome (CP/CPPS) is one of the most common medical conditions in urology with estimated prevalence of 2.2–13.8% in men from different societies [1–4]. The exact etiology of CP/CPPS is still not-completely recognized. Different reports referred to non-recognized bacterial infection, psychogenic factors, retrograde flow of urine in prostatic ducts or pelvic floor dysfunction as possible etiologies [5].

CP/CPPS could be a seen across all adult men regardless their age; however, it's more a diagnosis of young men [6] with presentation ranging from pain as the main complaint with voiding, psychogenic and sexual problems all affecting patients’ quality of life. The nature of sexual disorders is highly heterogeneous and many reports documented high prevalence of erectile dysfunction (30–50%) among CP/CPPS [7, 8]. It was even reported that CP/CPPS is an independent predictor of ED with an odds ratio of 3.62 [9].

The common pathophysiological pathways of sexual dysfunction and prostatic pain/voiding symptoms is not yet clear. Increased Rho-kinase activation and impaired nitric oxidase synthase in pelvic structure (including prostate and penis) may increase intraprostatic pressure and decrease smooth muscle relaxation of penile tissues causing prostatitis symptoms and erectile dysfunction, respectively. Also, autonomic hyperactivity, atherosclerosis and metabolic syndrome may play a main or a co-mechanism for prostate associated sexual dysfunction [10–12].

Based on previous pathways, PDE5-inhibitors could be expected to have a role in treatment of CP/CPPS. It was proven that tadalafil can down-regulate Rho-kinase activity. Rat model of chronic non-bacterial prostatitis had significantly suppressed pelvic pain and prostatic inflammation after tadalafil medications [13, 14]. Also, tadalafil can upregulate NO/cGMP resulting in reduced prostatic smooth muscles contractions [15, 16]. PDE5-inhibitors can also reduce atherosclerosis and inflammation by decreasing expression of various inflammatory markers [17].

In their clinical observation, Grimsley and colleagues found that known CP/CPPS patients prescribed PDE5-inhibitors for associated erectile dysfunction showed improvement in their prostatitis symptoms. They hypothesized that PDE5-inhibitors may mediate relaxation of prostatic duct smooth muscle which may reduce prostatic inflammation [18].

Clinically; few limited studies had investigated the outcome of PDE5-inhibitors in management of CP/CPPS, unfortunately, these studies mostly lacked control arm [19–22]. In this placebo-controlled clinical trial we aimed to evaluate the results of tadalafil treatment alone in management of non BPH-related CP/CPP patients.

## Patients and methods

### Study design

Our study run as double-blind placebo controlled clinical trial at urology department, Tanta University during the period from January 2019 till January 2022. The study was approved by our local review board and an informed written consent was given by each participant.

### Patient evaluation and selection

The study included male patients ≤ 45 years old with long history of CP/CPPS (≥ 1 year) who had recurrent/persistent symptoms after previous treatment with 4–6 weeks antibiotics and/or alpha blockers and who claimed complaints of erectile dysfunction. After careful history taking including all medications history, all patients were asked to fill Arabic translated and validated versions of NIH-CPSI and IIEF-5 forms [23, 24]. All patients underwent routine examination/digital rectal examination, laboratory evaluation, Meares–Stamey four-glass test, uroflowmetry and abdominal ultrasound evaluation.

Excluded patients were those with mild symptoms (total CPSI score ≤ 14), IIEF-5 ≥ 22, abnormal DRE or PSA values, history of uncontrolled diabetes mellitus, cardiac or nitroglycerine medications, ureteric/urinary bladder stones, urethral stricture or neurogenic disorders affecting lower urinary tract. Also, patients with renal or hepatic dysfunction, active urinary tract infection or bacterial prostatitis were excluded.

All selected patient were asked to stop any related medications (antibiotics, α-blockers, antimuscarinics, β_3_-agonists and NSIADs) for 2 weeks before start of treatment.

### Randomization and intervention

A total of 140 patients who fulfilled our criteria were randomized using random allocation software (RAS) to randomly distribute patients into two equal groups (70 for each). Tadalafil-group patients were given tadalafil 5 mg once daily at bedtime and placebo group (control-group) were given placebo starch tablet at bedtime for 6 weeks. Both patients and physician were blind regarding treatment given.

### Endpoint evaluation

Our primary endpoint was the evaluation of the 6th week’s CPSI domains score in comparison to baseline as well as to placebo with evaluation of clinically significant responders (improvement of ≥ 25% of total CPSI score). Our secondary endpoint was to find if the changes in CPSI domains scores are related to that of IIEF-5 scores.

### Sample size calculation

based on data from previous literatures, an expected reduction of 25% or 4–6 points in total CPSI scores at treatment arm or 3 points difference between both groups is expected to be clinically significant [25, 26]. With assumed SD of patients’ population around 5.5, significance level at 5% and power value of 80%; it was found that at least 54 patients were required to be included in each group to determine that difference.

### Statistical analysis

All data were presented as mean ± SD. For each patient, the change in CPSI score was *t*calculated as (∆CPSI = 6th week score-baseline CPSI score) where the negative values refer to symptoms’ improvement. Statistical analysis included two tailed paired/unpaired test to compare domains scores within each group or between both groups. Chi-square test was carried out to compare categorical variables in both groups. Pearson studies applied to define the correlation between ∆IIEF-5 score to ∆CPSI domains (both presented as percentage). For all statistics, *p* value was considered significant at *p* < 0.05.

## Results

**Fig. 1:**
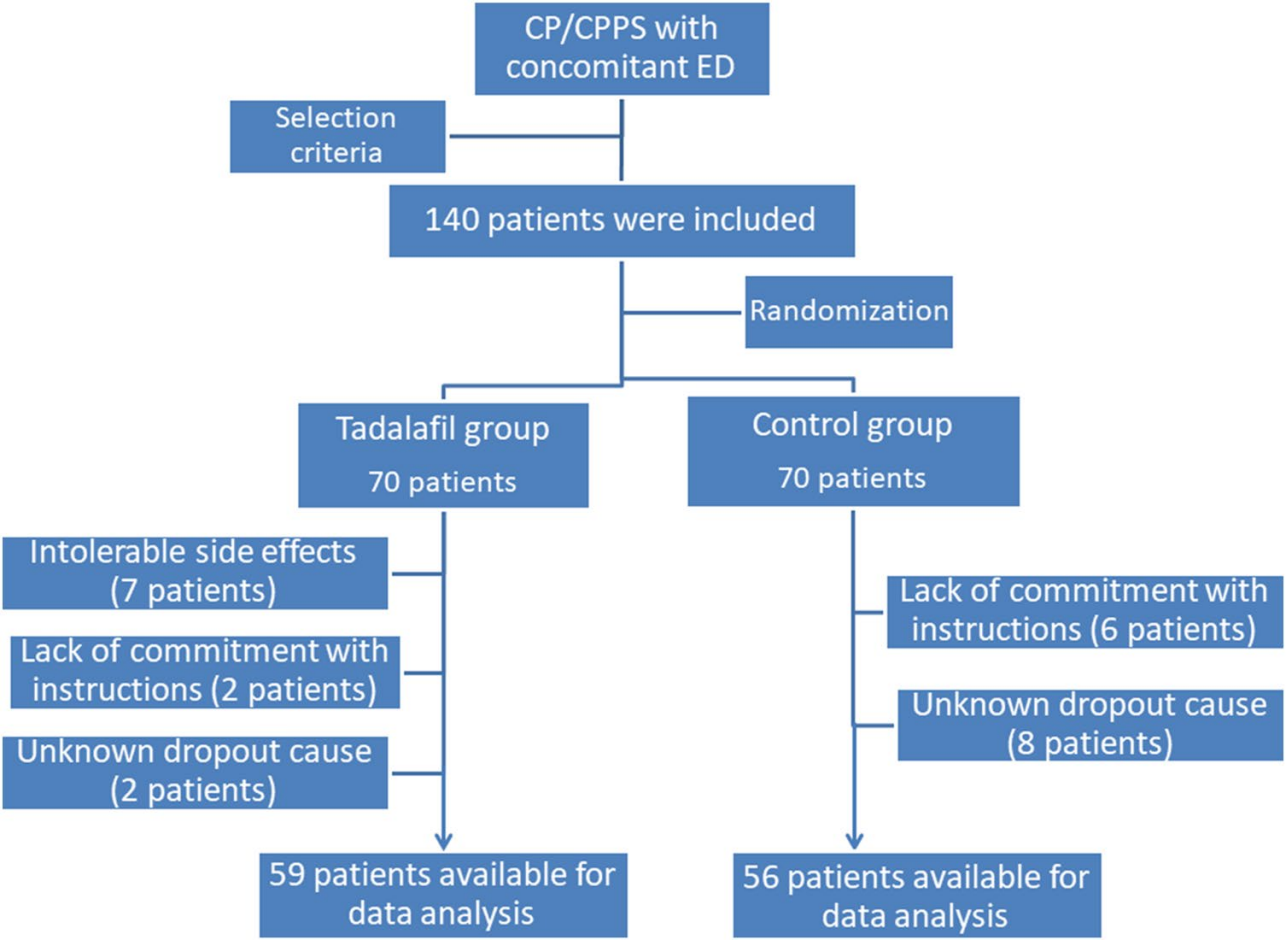
Consort flow chart

By the end of the study, 59 and 56 patients were available in groups I and II, respectively. It was noticeable that 7 patients in tadalafil-group (10%) reported medicine related side effect including muscle ache, headache and flushes during the 1st week and didn’t continue the study protocol (Fig. 1).

Both groups were homogenous regarding history and clinical data at presentation. History of previous interrupted PDE5-inhibitors usage was also not statistically different between both groups (Table 1).

**Table 1:** Demographic data of both groups

	Tadalafil-group	Control-group
Age		
Range	32–45	30–45
Mean ± SD	39.9 ± 3.9	39.7 ± 4.7
Median	41	40.5
Severity of symptoms (CPSI score)		
Moderate (15–29)	45	46
Severe (30–43)	14	10
Duration of symptoms (months)		
Range	12–48	12–42
Mean ± SD	21.9 ± 7.77	21.11 ± 8.01
Median	20	18
Previous PDE5-inhibitors therapy (*n*.)		
Tadalafil 5 mg OD	3	6
Tadalafil 20 mg PRN	6	7
Other PDE5-inhibitors	21	18
PSA value (ng/ml)		
Range	0.5–2.9	0.5–2.8
Mean ± SD	1.3 ± 0.6	1.3 ± 0.5
Prostate volume (ml)		
Range	16–33	17–32
Mean ± SD	23.6 ± 3.8	23.5 ± 3.6

The baseline IIEF-5 and CPSI domains scores were not statistically different in both groups (*p* > 0.05). Compared to baseline, tadalafil-group patients showed significant reduction of all CPSI domain scores after treatment (*p* < 0.05). Regarding post-treatment results in both groups; only urinary, Qol and total CPSI domains were significantly better (*p* < 0.05) but pain score did not (*p* = 0.07) (Table 2).

**Table 2:** Baseline and post treatment results in both groups

	Tadalafil group (59 patients)		Control-group (56 patients)	
	Baseline score	6th week score	Baseline score	6th week score
Pain				
Range	6–20	3–19	5–20	4–20
Mean ± SD	12.14 ± 3.57	10.42 ± 3.55*	12.04 ± 3.88	11.71 ± 3.9
Median	12	10	11.5	11.5
Urinary				
Range	2–9	1–8	1–9	1–9
Mean ± SD	6.08 ± 1.53	4.2 ± 1.72*^,Ұ^	6.04 ± 1.62	5.93 ± 1.73
Median	6	4	6	6
Qol				
Range	3–9	2–9	4–9	3–9
Mean ± SD	6.22 ± 1.76	4.47 ± 1.64*^,Ұ^	6.23 ± 1.25	6.14 ± 1.46
Median	6	4	6	6
Total				
Range	15–37	9–31	16–34	13–35
Mean ± SD	24.21 ± 5.05	19.1 ± 5.26*^,Ұ^	24.3 ± 4.51	23.79 ± 5.2
Median	23	18	24	24
IIEF-5				
Range	13–21	16–25	11–21	10–23
Mean ± SD	17.6 ± 2.2	21 ± 1.8*^,Ұ^	17.2 ± 3.04	17.46 ± 3.56
Median	17	21	18	18

*Statistically significant when compared to base line value, paired *t* test (*p* < 0.05), ^Ұ^Statistically significant when compared to 6th week values, unpaired *t* test, Welch corrected (*p* < 0.05)

**Fig. 2:**
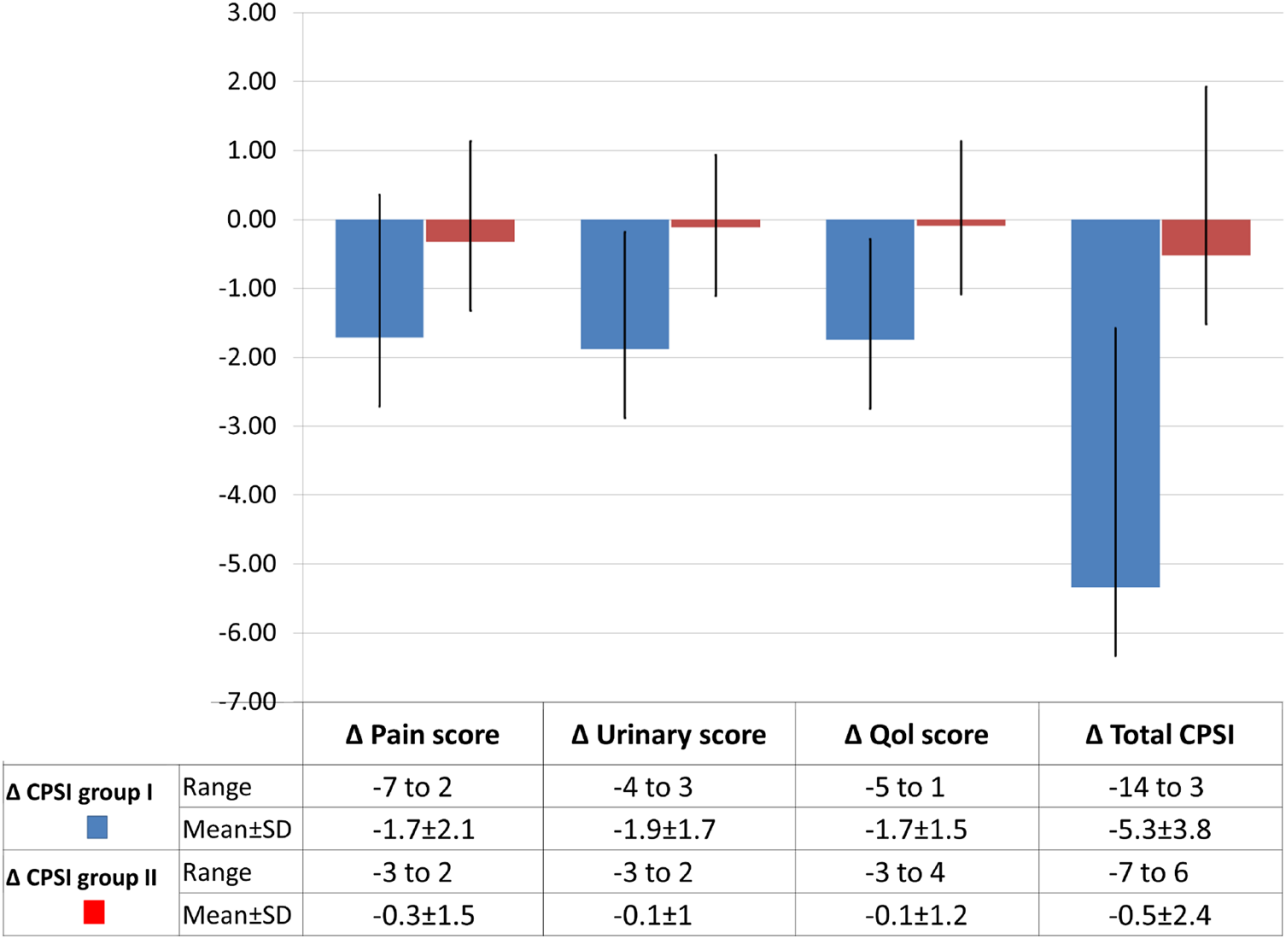
Changes of CPSI (∆CPSI) domains scores in both groups

Changes in CPSI domains scores (∆CPSI) were greater in tadalafil-group than control-group, *p* < 0.05, (Fig. 2). The clinically significant improvement (≥ 25% reduction from baseline scores), was seen in only 30 patients (50.8%) in tadalafil-group compared to 3 patients (5.4%) in control-group (*x*^2^ = 26.88, *p* < 0.05).

**Fig. 3:**
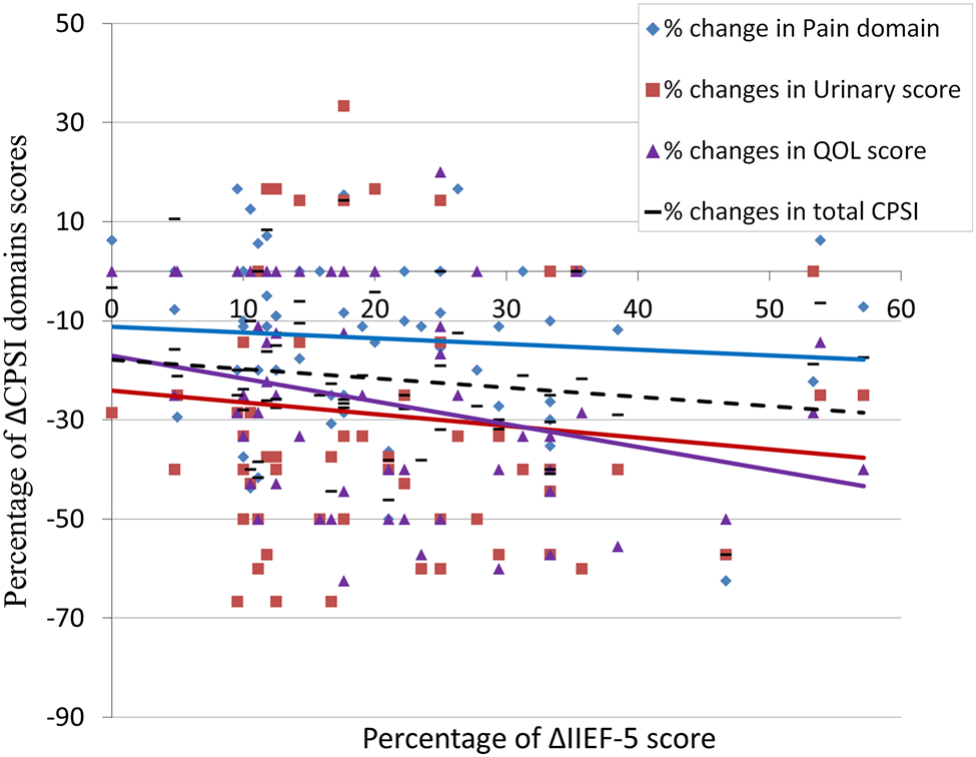
Relation of ΔCPSI domain score to ΔIIEF-5 score (both presented as percentages)

In tadalafil-group, there was a weak but significant correlation between percentage of changes in Qol domain and that of IIEF-5 scores (*r* = − 0.28, *p* < 0.05). For other CPSI domains changes, that correlation was insignificant (*r* = − 0.09, − 0.1 and − 0.16 respectively, *p* > 0.05) (Fig. 3).

## Discussion

Traditionally, we used antibiotic, anti-inflammatory, and α-blockers either alone or in combination to treat CP/CPPS. Although widely used, there are weak evidences to guarantee satisfactory outcome [5].

Several studies showed that tadalafil 5 mg once daily can significantly improve LUTS independent of severity or improvement in ED [27]. In addition to its use in BPH, the literatures carry good experimental and pre-clinical supports for using PDE5-inhibitors in management of prostatitis [13–15]. Clinically, there are few studies discussing its role in CP/CPPS. Unfortunately, most of these studies either lacked control arm or run in BPH associated CP/CPPS or in combination with other medications [19–21].

In a non-controlled 3 months follow up study, Benelli et al. reported that tadalafil significantly improved all NIH‐CPSI domains scores; this reduction was noticed as soon as one month post treatment and was mainly seen in pain score (reduced from 13.7 ± 3.7 to 5.4 ± 2.2) as well as total, micturition and QOL CPSI sub-scores (decreased from 27.6 ± 4.2, 5.2 ± 3.8 and 10.1 ± 1.9 to 8.8 ± 3.2, 1.4 ± 1.7 and 1.9 ± 0.8 respectively). Further decline was seen by the 2nd and 3rd months [28].

To standardize the outcome of CP/CPPS clinical treatment trials, many researchers suggest ≥ 25% reduction in post treatment total CPSI score to be clinically significant and perceived by patients as improvement [25, 26, 29].

Although Benelli’s study didn’t report those clinically significant responders, but the very big difference in pre/post treatment median scores suggest that there was a good number of clinically significant responders (unlike our study where only 50.8% developed clinical response). The small number of included patients in their study (14 patients) may not give enough study power to guarantee the results. Also, the selection criteria (only 3 months symptoms duration) may explain that great response.

Many clinical trials studied tadalafil effect in CP/CPPS associated with BPH. Due to overlapping of CP/CPPS urinary symptoms and those of BPH/LUTS, the reduction in pain subscore may be considered as the valuable parameter to detect improvement. Hiramatsu et al. reported that 3 months of tadalafil treatment can significantly reduce pelvic pain associated with LUTS/BPH. For the subgroup with severe pain (domain score ≥ 4), mean improvement in total CPSI and pain sub‐scores were -10.0 ± 7.8, and − 4.4 ± 4.5. Also, there was a significant reduction in micturition and QOL domains (− 2.5 ± 3.0 and − 3.1 ± 2.7) [22]. In spite of adding tadalafil to α-blockers, Matsukawa et al. showed more limited but still significant CPSI score changes (changes in CPSI total, pain, urinary and QOL subscore were − 4.6 ± 4.4, − 1.5 ± 2.1, − 1.4 ± 1.4 and 1.8 ± 2.2, respectively). Only four patients (8.9%) showed ≥ 50% improvement in their CPSI score [20]. it's to be noted that both studies run in elderly BPH population and lacked control arm.

In Pineault et al. study, 25 patients (mean age 44.4 ± 12.9 years) were treated with tadalafil 5 mg daily for a mean duration of 1.3 ± 1.6 years. Although lacked control arm, they concluded that daily use of tadalafil for long time was associated with significant decreases in CPSI total, pain, urinary symptom and quality of life scores (− 12.8 ± 9.5, − 6.1 ± 4.1, − 2.4 ± 2.1 and − 4.5 ± 3.9, respectively) [21]

The significant changes in CPSI scores, compared to baseline, in our study are found to be in accordance with the previously mentioned reports but with much limited changes. That limited changes of CPSI may be explained as our study included younger ages with longer duration of moderate/severe complaint or may be attributed to shorter study duration.

To our knowledge, this is the first placebo controlled clinical trial comparing tadalafil effect to placebo in young adults with refractory CP/CPPS. Unlike other CPSI domain scores, pain subscore didn't achieve statistically significant improvement when compared to placebo (*p* > 0.05). The lack of previous controlled studies makes it difficult to explain the cause. The chronicity of symptoms or the short period of study could be possible factors.

In addition to treatment induced changes on CPSI score, our results support the well-known idea of PDE5-inhibitors as an effective treatment of ED whatever the etiology especially in mild/moderate cases [30]. Tadalafil significantly improved IIEF-5 in CP/CPPS patients compared to placebo. That effect could be explained as most of CP/CPPS patients had non severe form of ED.

Due to poor long-term treatment success, CP/CPPS can impact quality of life (Qol) and can contribute to erectile dysfunction [31, 32]. Vice-versa, erectile dysfunction is strongly associated with a negative impact on the QOL [33]. Based on this two-ways relation, Qol changes secondary to tadalafil-induced improvement in sexual function could, theoretically, affect our results regarding CPSI. However, our correlation statistics found only a weak negative but significant correlation between changes in IIEF-5 score and CPSI Qol domain (*r* = − 0.28, *p* < 0.05). That relation was not seen between IIEF-5 changes and CPSI total, pain or micturition scores.

Limitations in our study included the short duration. CP/CPPS is considered a chronic inflammatory process and longer treatment protocols are usually advised. However, even with 6 weeks duration, our results are promising for future longer duration studies.

Another limitation was our selection criteria; tadalafil is currently not approved for management of CP/CPPS, so we used it only in those with CP/CPPS concomitant ED. The overlapping effect of tadalafil-induced IIEF-5 score changes and Qol subscore was reported to be weak but significant which may affect our conclusion. Further controlled trials in CP/CPPS patients without co-morbid ED may be required.

## Conclusion

In this placebo-controlled clinical trial, patient with moderate/severe CP/CPPS managed by tadalafil 5 mg once daily for 6 weeks showed significant improvement of all CPSI domains (pain, micturition, Qol and total scores) compared to baseline. When compared to placebo, CPSI pain domain didn’t achieve such significant improvement unlike all other domains which were significantly better. The changes in pain, urinary and total scores were not correlated with the change in IIEF-5 score. The fact that only 50.8% of patients get clinically significant improvement (≥ 25% reduction in total score) suggests this medicine may be considered in combination with other treatment protocols in the future studies.

## Abbreviations

CP/CPPS: Chronic prostatitis associated chronic pelvic pain syndrome
CPSI: Chronic Prostatitis Symptoms Index.
IIEF-5: 5-Items version of International Index of erectile function
∆CPSI: Changes in Chronic Prostatitis symptoms Index Score
∆IIEF-5: Changes in International Index of erectile function 5 items scores
LUTS: Lower urinary tract symptoms
ED: Erectile dysfunction

## Declarations

### Research involving humans and/or animals

This research involved human participants.
