# Correction to: Tadalafil monotherapy in management of chronic prostatitis/chronic pelvic pain syndrome: a randomized double-blind placebo controlled clinical trial

**DOI:** 10.1007/s00345-022-04128-7

**Published:** 2022-08-18

**Authors:** Ahmed M. Tawfik, Mohammed H. Radwan, Mohammed Abdulmonem, Mohammed Abo-Elenen, Samir A. Elgamal, Mohammed O. Aboufarha

**Affiliations:** 1 Urology Department, Faculty of Medicine, Tanta University, Tanta, Egypt; 2 Urology Department, Faculty of Medicine, Zagazig University, Zagazig, Egypt

## Correction to: World Journal of Urology 10.1007/s00345-022-04074-4

There is a mistake in the top cells of Table 2 in the original publication of the article. The top cells should be shifted one column to the right to make it inline with the corresponding data. The correct Table 2 is given below:

**Table 2:** Baseline and post treatment results in both groups

		Tadalafil group (59 patients)		Control-group (56 patients)	
		Baseline score	6th week score	Baseline score	6th week score
Pain	Range	6–20	3–19	5–20	4–20
	Mean ± SD	12.14 ± 3.57	10.42 ± 3.55 ^*^	12.04 ± 3.88	11.71 ± 3.9
	Median	12	10	11.5	11.5
Urinary	Range	2–9	1–8	1–9	1–9
	Mean ± SD	6.08 ± 1.53	4.2 ± 1.72^*,Ұ^	6.04 ± 1.62	5.93 ± 1.73
	Median	6	4	6	6
Qol	Range	3–9	2–9	4–9	3–9
	Mean ± SD	6.22 ± 1.76	4.47 ± 1.64^*,Ұ^	6.23 ± 1.25	6.14 ± 1.46
	Median	6	4	6	6
Total	Range	15–37	9–31	16–34	13–35
	Mean ± SD	24.21 ± 5.05	19.1 ± 5.26^*,Ұ^	24.3 ± 4.51	23.79 ± 5.2
	Median	23	18	24	24
IIEF-5	Range	13–21	16–25	11–21	10–23
	Mean ± SD	17.6 ± 2.2	21 ± 1.8^*,Ұ^	17.2 ± 3.04	17.46 ± 3.56
	Median	17	21	18	18

*: Statistically significant when compared to base line value, paired *t* test (*p* < 0.05)

^Ұ^: Statistically significant when compared to 6th week values, unpaired *t* test, Welch corrected. (*p* < 0.05)

The original article has been corrected.

